# Supplementary material for: Splice-Junction-Based Mapping of Alternative Isoforms in the Human Proteome
Source: Cell Rep. Author manuscript; Available in PMC 2020 Jan 15. (PMC6961840; doi:10.1016/j.celrep.2019.11.026)
Supplement: 2 [file NIHMS1546469-supplement-2.zip › DF1/PXD010154/Thyroid/Thyroid_19_DESI2_HTKLDERIYLIHWNWSFSFR.pdf]

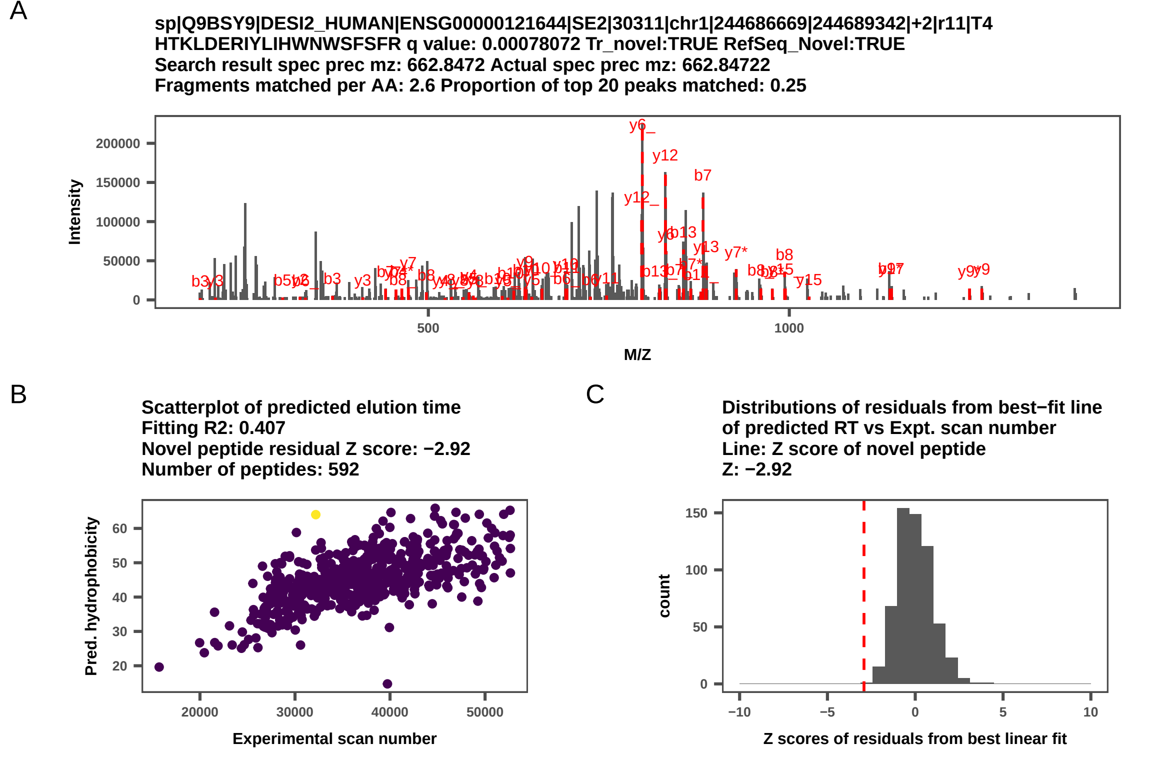
A
sp|Q9BSY9|DESI2_HUMAN|ENSG00000121644|SE2|30311|chr1|244686669|244689342|+2|r11|T4
HTKLDERIYLIHWNWSFSFR q value: 0.00078072 Tr_novel:TRUE RefSeq_Novel:TRUE
Search result spec prec mz: 662.8472 Actual spec prec mz: 662.84722
Fragments matched per AA: 2.6 Proportion of top 20 peaks matched: 0.25
Intensity
200000
150000
100000
50000
0
500
1000
M/Z
B
Scatterplot of predicted elution time
Fitting R2: 0.407
Novel peptide residual Z score: −2.92
Number of peptides: 592
Pred. hydrophobicity
60
50
40
30
20
20000
30000
40000
50000
Experimental scan number
C
Distributions of residuals from best−fit line
of predicted RT vs Expt. scan number
Line: Z score of novel peptide
Z: −2.92
count
150
100
50
0
−10
−5
0
5
10
Z scores of residuals from best linear fit
